# Supplementary figures and images for: Mining ancient microbiomes using selective enrichment of damaged DNA molecules
Source: BMC Genomics. 2020 Jun 26;21:432. doi: 10.1186/s12864-020-06820-7 (PMC7318760; doi:10.1186/s12864-020-06820-7)

**A.**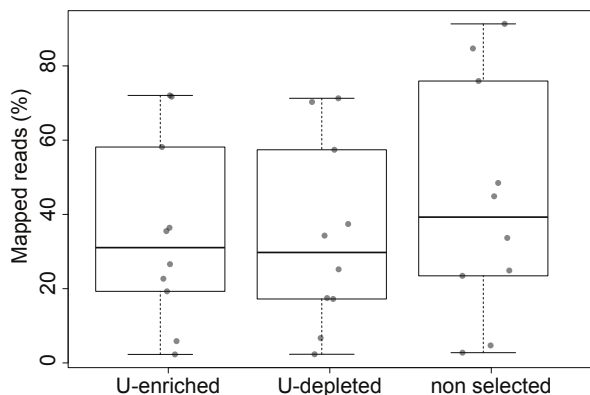**B.**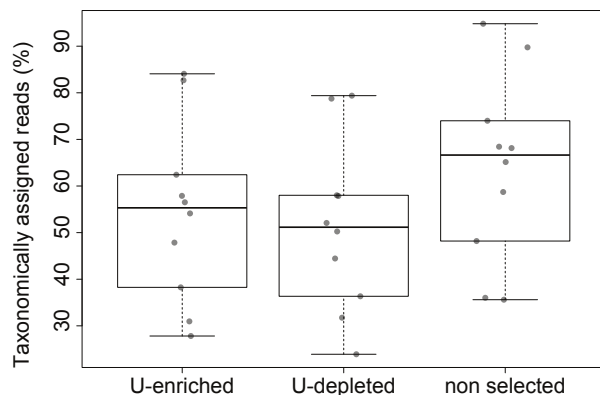**C.**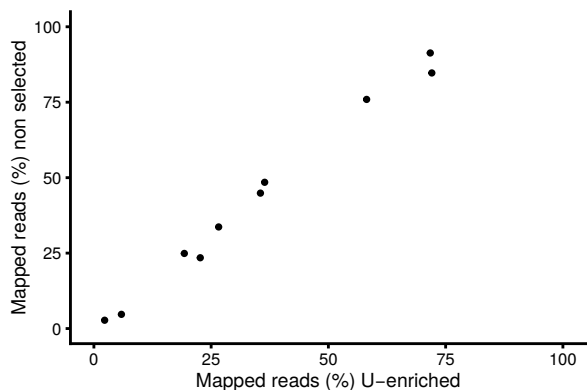**D.**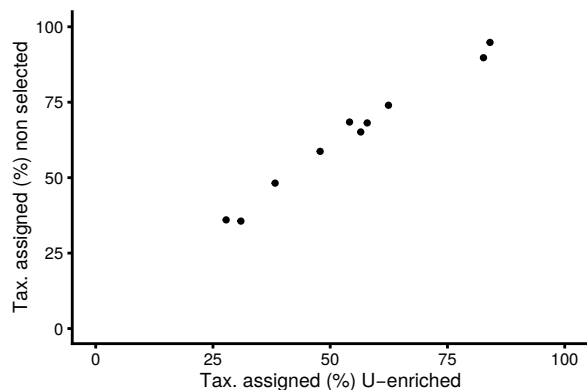**E.**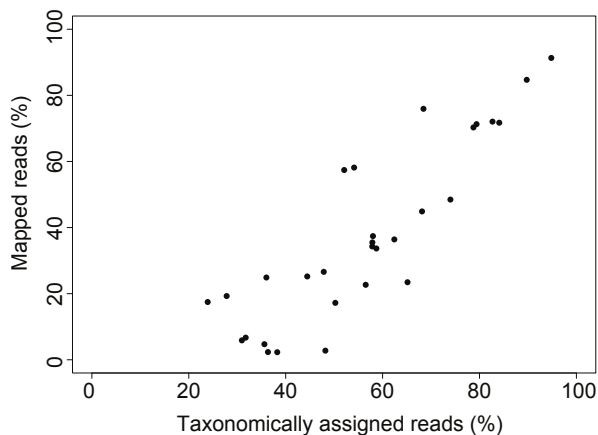

Supplement: Supplementary file 1 — Additional file 1: Figure S1. Mapped and taxonomically assigned reads of plant historical specimens. A. Distributions of percentage of mapped reads for non-selected and U-selected libraries (U-enriched and U-depleted fractions). B. Distributions of percentage of taxonomically assigned reads for non-selected and U-selected libraries (U-enriched and U-depleted fractions). C. Correlation of the percentage of mapped reads between the U-enriched and the non-selected library D. Correlation of the percentage of taxonomically assigned reads between the U-enriched and the non-selected library E. Relation between percentages of mapped and taxonomically assigned reads from U-selected libraries (U-enriched fraction). [file 12864_2020_6820_MOESM1_ESM.pdf]

**A.**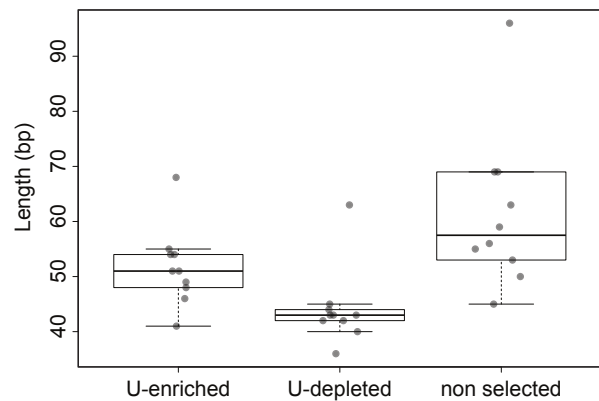**B.**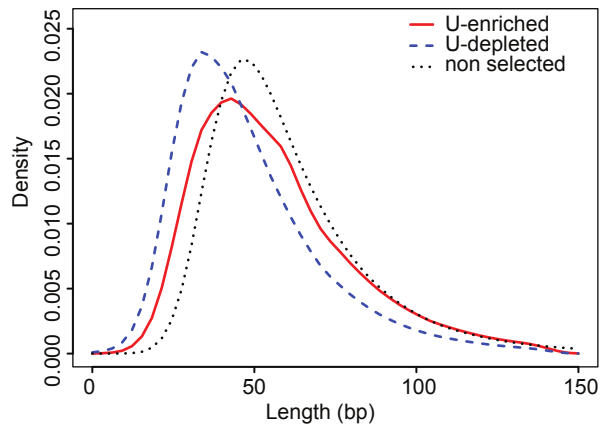**C.**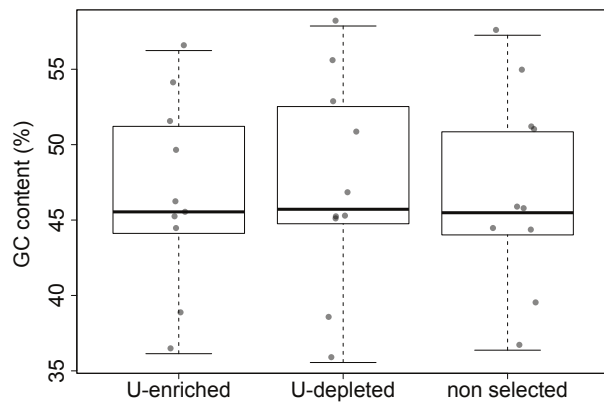

Supplement: Supplementary file 2 — Additional file 2: Figure S2. Length and GC content of plant historical specimens. A. Distributions of mean length for non-selected and U-selected libraries (U-enriched and U-depleted fractions). Median values are denoted as black lines and points show the original value for each individual sample. B. Length distribution of Arabidopsis thaliana sample NY1365375 for a non-selected and U-selected library (U-enriched and U-depleted fractions). C. Distributions of mean GC content for non-selected and U-selected libraries (U-enriched and U-depleted fractions). [file 12864_2020_6820_MOESM2_ESM.pdf]

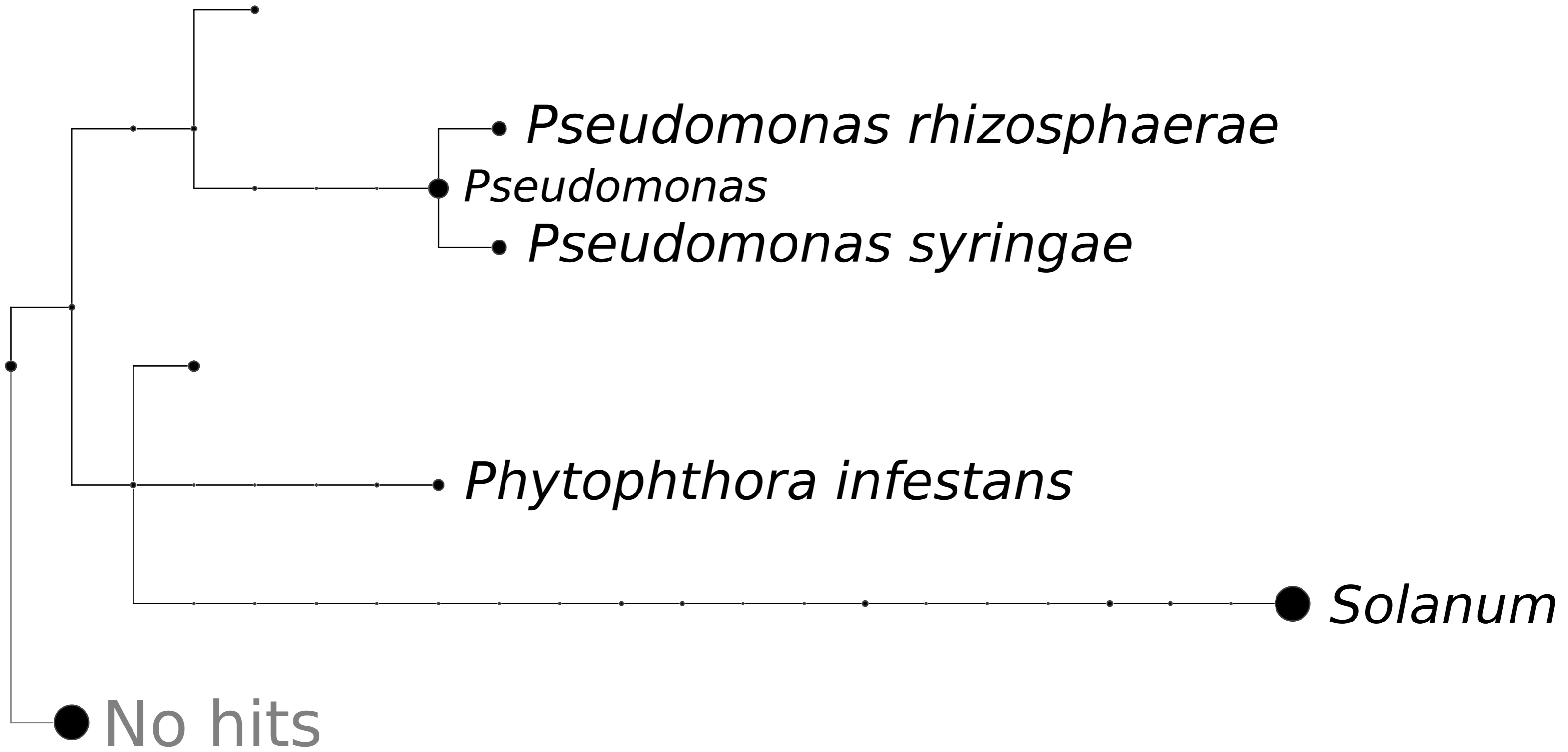

Supplement: Supplementary file 3 — Additional file 3: Figure S3. Taxonomic tree of reads from a Solanum tuberosum library assigned to different taxonomic levels. The size of the circle represents the amount of reads assigned to the node displayed in the tree or to any taxonomic level below it. Reads assigned to some species Phytophthora infestans, Pseudomonas syringae and Pseudomonas rhizosphaerae, as well as the genera Pseudomonas and Solanum are named in the taxonomic tree. [file 12864_2020_6820_MOESM3_ESM.pdf]

**A.**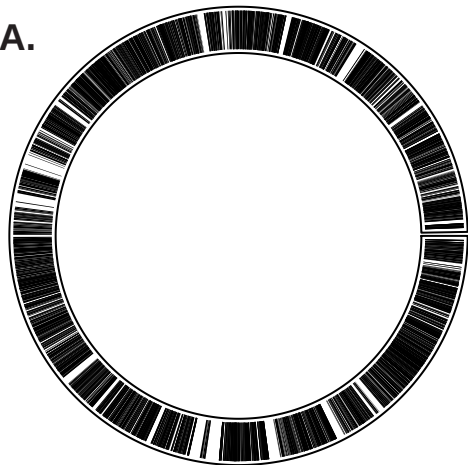*P. syringae***B.**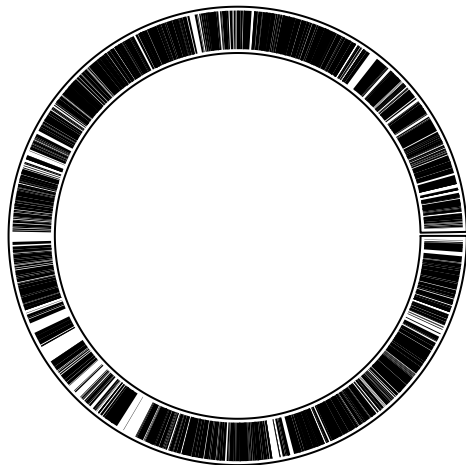*P. rhizosphaerae***C.**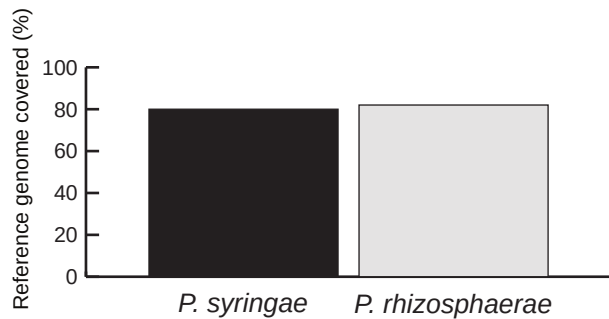

Supplement: Supplementary file 4 — Additional file 4: Figure S4. Analysis of de novo assembled Pseudomonas contigs from a Solanum tuberosum sample. Contig coverage of Pseudomonas syringae (A.) and Pseudomonas rhizosphaerae (B.). The circles represent each circular bacterial reference genome, and the black lines depict genomic regions covered by alignments of de novo assembled contigs. C. Percentage of reference genome of P. syringae and P. rhizosphaerae covered by de novo assembled contigs from A. and B., respectively. [file 12864_2020_6820_MOESM4_ESM.pdf]

**A.**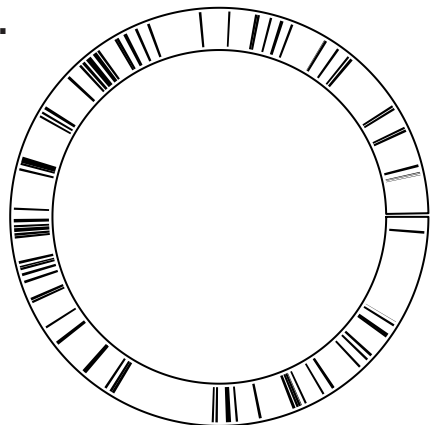*P. syringae***B.**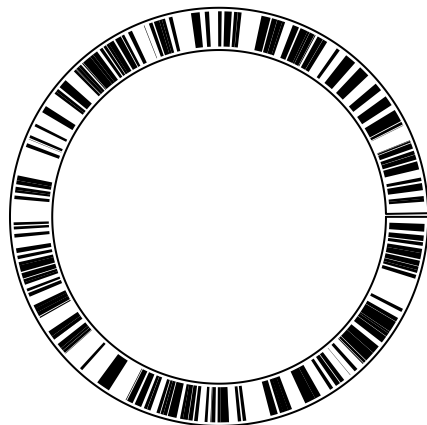*P. rhizosphaerae***C.**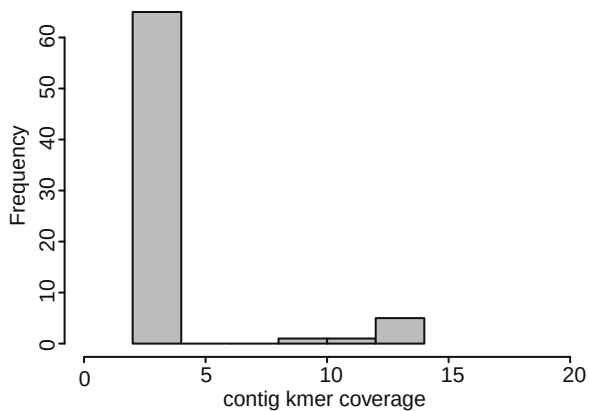**D.**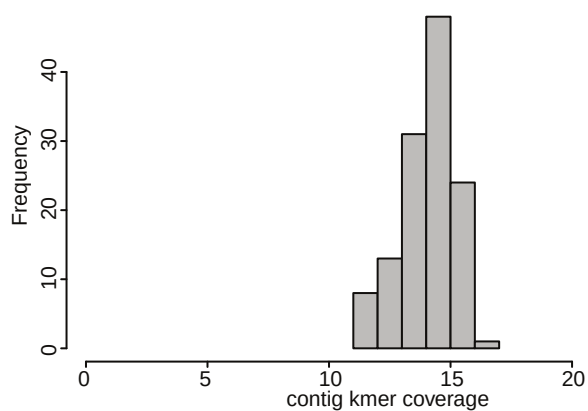

Supplement: Supplementary file 5 — Additional file 5: Figure S5. Analysis of uniquely mapped Pseudomonas contigs from a Solanum tuberosum sample. Uniquely mapped contig coverage of Pseudomonas syringae (A.) and Pseudomonas rhizosphaerae (B.). The circles represent each circular bacterial reference genome, and the black lines depict genomic regions covered by alignments of uniquely mapped contigs. Histograms of contig k-mer coverage from de novo assembled contigs uniquely mapping to P. syringae (C.) and P. rhizosphaerae (D.). [file 12864_2020_6820_MOESM5_ESM.pdf]
